# Preclinical Studies on Mesenchymal Stem Cell-Based Therapy for Growth Plate Cartilage Injury Repair

**DOI:** 10.4061/2011/570125

**Published:** 2011-07-26

**Authors:** Rosa Chung, Bruce K. Foster, Cory J. Xian

**Affiliations:** ^1^School of Pharmacy and Medical Sciences, Sansom Institute for Health Research, University of South Australia, City East Campus, G.P.O Box 2471, Adelaide, SA 5001, Australia; ^2^Discipline of Physiology, School of Medical Sciences, University of Adelaide, Adelaide, SA 5005, Australia; ^3^Department of Orthopaedic Surgery, Women's and Children's Hospital, North Adelaide, SA 5006, Australia

## Abstract

In the last two decades, there has been a strong interest in searching for biological treatments for regeneration of injured growth plate cartilage and prevention of its bony repair. Various means have been tried, including implantation of chondrocytes, mesenchymal stem cell (MSC), together with exogenous growth factor and scaffolds, and gene therapy. However, with the lack of success with chondrocytes, more research has focussed on MSC-based treatments. In addition to circumvent limitations with MSC-based treatments (including cell harvest-associated morbidity, difficulties/time/cost involved in MSC isolation and *ex vivo* expansion, and potential disease transmission), mobilising endogenous MSCs to the growth plate injury site and enhancing *in situ* regeneration mechanisms would represent an alternative attractive approach. Further studies are required to investigate the potential particularly in large animal models or clinical setting of the *ex vivo* MSC approach and the feasibility of the endogenous MSC *in situ* approach in growth plate regeneration.

## 1. Introduction

Situated at the ends of all long bones, the growth plate is solely responsible for the lengthening of long bones. However, being of a cartilaginous nature, the growth plate is highly susceptible to injuries. Depending on the severity and location, often these injuries are often repaired undesirably by bony repair tissue (also known as a bone bridge formation) which in turn often results in orthopaedic conditions such as limb length discrepancies and bone angulation deformities. As the current methods of correcting growth plate injury-induced bone growth defects are surgically based, highly invasive and not always successful, increasing interest has been shown towards the development of biological treatments which aim to promote growth plate cartilage regeneration and prevent the faulty bony repair. However, although a myriad of studies have investigated potential therapeutic effects of tissue-, chondrocyte-, growth factor-, or mesenchymal stem cells-(MSC-) based approaches in repairing injured growth plate with different degrees of success, currently there is not a biological therapy clinically available that can induce growth plate regeneration. This paper attempts to summarise previous and current research investigating therapeutic potentials of various biological materials or approaches with a particular focus on MSC-based therapies in attempt to induce growth plate cartilage regeneration.

## 2. The Growth Plate Cartilage

Children's long bones contain a large cartilaginous region known as the growth plate (epiphyseal plate) which is responsible for the longitudinal growth of that particular long bone, through chondrocyte proliferation, hypertrophy, apoptosis, cartilage matrix synthesis, mineralization, and vascularisation [1–3]. The area of this cartilaginous region significantly decreases as the young person gets older and it closes when the maximum growth of the long bone is achieved. The region directly under the growth plate is called the metaphysis which is where the mineralised growth plate cartilage is being replaced by bone, a process called endochondral ossification [4]. Thus, endochondral ossification bone lengthening is via a two-step process that involves growth plate cartilage scaffold formation and the differentiation and function of bone-forming cells osteoblasts to initiate bone formation in the metaphysis [1, 5].

## 3. Growth Plate Injury and Current Treatments

**Figure 1:**
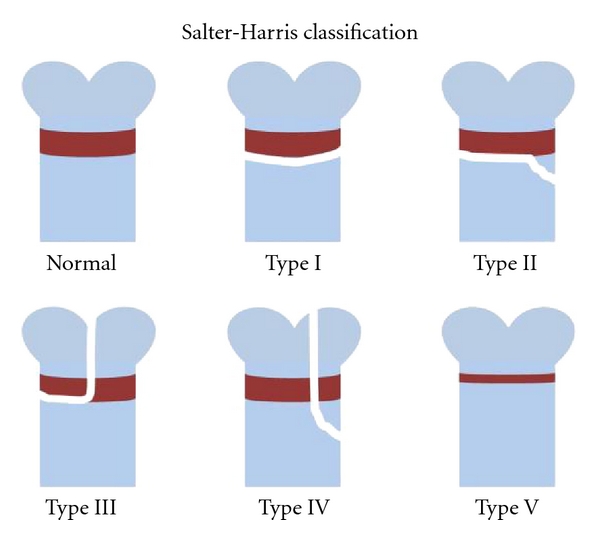
The Salter-Harris classification system. Types I and II fractures do not affect the epiphyseal blood supply. On the other hand, types III, IV, and V do disrupt the blood supply and will more than often result in undesirable bony repair tissue-causing problems of angulations and growth arrest.

Due to accidents in sports and play, skeletal fractures are common in children, with up to 50% children of 5–18 years old experiencing a bone fracture [6]. Since the growth plate is the least rigid region of the long bone, its injuries are common, and it has been estimated that around 20% childhood bone fractures involve growth plate [7]. The Salter-Harris classification system has been used to distinguish the different types of growth plate injuries and the relationship between the characteristics of the fractures and their prognoses (Figure 1) [1, 8–10]. Current literature indicates that the most common type of growth plate fractures occurring in the distal tibiae of younger children is type II (around 40%), which in most cases has a reasonably good prognosis as the cells responsible for interstitial growth of the growth plate as well as the epiphyseal blood supply remain undisturbed [10–12]. Other types of fractures, types III, IV, and V, however, may/will all result in bony formation at the injured site [13]. It has been estimated that in up to 30% of all children with growth plate-related injuries, undesirable bony repair, and bone bridge at the injury site hinder normal growth of the developing long bone in the affected limb [14, 15], which results in significant orthopaedic problems such as limb length discrepancy and bone angulation deformity [15, 16].

Due to the significant orthopaedic problems resulting from growth plate injuries, many previous studies have looked at different ways of correcting growth plate injury-induced defects as well as preventing the bony repair [17]. The type of treatment for growth plate injuries is largely dependent on the age of the patient as well as the severity and type of injury sustained [18]. Surgical intervention is usually needed only if the patient is quite young and significant growth remains. If the injury only results in a very slight length discrepancy, it is often fixed through the use of a shoe lift, and in most cases the patient must cease using the affected limb for a period of time in order to prevent orthopaedic problems, such as angular deformity, from occurring. An already established angular deformity is commonly corrected with a wedge osteotomy [19–21]. On the other hand, larger limb length discrepancies require bone lengthening or bone shortening procedures [22–24]. The most common way of correcting larger limb length discrepancies is through a surgical and lengthening procedure which surgically create a fracture at the diaphysis and then gradually lengthens the injured limb to match the growth of the unaffected limb using a large external frame (*Ilizarov* frame) placed around the affected limb [20, 23, 25]. As effective as this method of treatment is, the downside is that the procedure is highly invasive, painful and lengthy. As only a limited amount of lengthening can be done at a time, the patient often requires the procedure several times throughout adolescence until skeletal maturity is reached. Furthermore, complications arising from pin site infections, further fractures, dislocation, and compartment syndromes make this procedure even more difficult [26]. More recently, another technique has been introduced which can be used to lengthen the affected limb involving the use of an implantable and programmable distraction internal nail known as “Fitbone” for adolescents who have reached maximal growth [27]. Fitbone eliminates the need for an external fixator and hence has the potential to reduce pain and the risk of infections occurring within the treatment site.

Sometimes, an established bone bridge can be surgically removed for correction of growth defects. In order to prevent growth arrest and angulation deformity from recurring, the defect site can be filled with transplanted fat, muscle, polymeric silicone, bone wax, and bone cement as interposition materials [17]. This procedure is called the Langenskiold method [28]. However, all of these available treatments so far are extremely invasive, time consuming, and often ineffective. Currently, much interest has been drawn in finding a better treatment (particularly by a preventative biological approach) to prevent and/or correct problems associated with bony bridge formation. In particular, in more recent times, more research has focussed on utilising tissue engineering and the use of mesenchymal stem cells (MSC) for the regeneration of growth plate cartilage.

## 4. Previous Attempts with Chondrocyte/Cartilage Transplantation

This void or deficiency of a biological treatment for growth plate injuries has instigated many medical scientists and clinicians to find a potential biological therapy which is able to prevent the bony repair at the injured growth plate and hence thwart the serious orthopaedic problems associated with this condition. Ideally, a successful therapy would have the ability to regenerate the growth plate cartilage so that the long bone is able to grow with minimal disruption minimising any angulation and/or growth arrest of the affected limb. However, as with any cartilaginous structures the transphyseal growth plate injuries are very hard to heal to the original state as chondrocytes are very difficult to regenerate [29, 30]. 

Allogeneic and autologous chondrocyte transplantations are one potential approach to overcome this problem, and both methods of chondrocyte transplantation have previously been utilised or trialled for articular cartilage or growth plate repair studies. Allogeneic chondrocyte transplantation involves the removal of healthy chondrocytes from one source followed by the *ex vivo* expansion and finally the replantation of the expanded chondrocytes into another individual (of the same species) [31]. However, the disadvantages of this procedure involve the risk of disease transmission between the two individuals. Alternatively, autologous chondrocyte transplantation involves the direct harvest of healthy chondrocytes (often from the knee) which are then cultured and expanded *ex vivo*; unlike the allogeneic approach, the chondrocytes are implanted back into the patient at the location of the defect, therefore, eliminating any risks of disease transmission [32]. Nevertheless, the disadvantage of this method is the time frame taken to collect, expand, and reimplant the chondrocytes, which has been estimated 3 weeks [33]—by which time, in the case of growth plate injury—a bone bridge has already started to form, thus eliminating this autologous chondrocyte transplantation approach being feasible for growth plate regeneration. 

Although there have been many successful studies which have used the allogeneic and autologous chondrocyte transplantation approach for articular cartilage regeneration, very few studies have been performed on growth plate injury models. One earlier study, Bentley and Greer [34] found some success when allogeneic chondrocytes (collected from the growth plate) were delivered into the growth plate injury site of White New Zealand rabbits. This study reported that chondrocytes filled the defect and were able to form columns. In addition, although there were signs of endochondral ossification at the base of the injury site, no rejection of the implanted chondrocytes occurred [34]. However, one study, using a large animal (sheep) tibial growth plate injury model, attempted transplanting chondrocytes directly into the growth plate injury site and did not produce any successful outcomes in preventing the bony bridge formation [35]. Hence, this highlights the unlikelihood of achieving successful growth plate cartilage regeneration with this chondrocyte transplantation approach. 

## 5. Recent Attempts with MSC-Based Growth Plate Cartilage Repair

Due to the limitations associated with chondrocyte transplantation including instability during expansion and donor tissue availability as well as outcome success [36], an alternative cell source, that has been heavily investigated, has been the stem cells. Being of an undifferentiated type, embryonic stem cells hold great potential in differentiation and successful tissue engineering; however, the myriad of ethical and potential health risks and dilemmas involved with their use deem them almost inaccessible [37]. On the other hand, adult mesenchymal stem cells (MSC) are renewable, undifferentiated pluripotent cells which are also capable of differentiating into many different cell types [38] such as cartilage, bone, and fat cells. 

MSCs are abundant and have been successfully isolated from many sources including bone marrow [39, 40], periosteum [41–43], trabecular bone [44, 45], adipose tissue [46–48], skeletal muscle [49, 50], and synovium [51–53]. Due to their pluripotency, abundance and accessibility, bone marrow-derived MSCs have made a particularly attractive source for use in articular and growth plate cartilage regeneration [5, 22, 39, 54]. Additionally, an *in vivo* study done by Park et al. [55] showed that MSCs derived from bone marrow and perichondrium/periosteum were more successful at forming hyaline cartilage than from those MSCs derived from other sources such as adipose tissue [55].

Although bone marrow-derived MSCs make up a small proportion of total marrow nucleated cells, they can be easily isolated and expanded with high efficiencies [36]. A plethora of bone marrow-derived MSC related studies have demonstrated the ability of MSCs to differentiate *in vitro* into multiple cell lineages depending on defined culture conditions including differentiation into chondrocytes [40, 54, 56]—making them an ideal candidate for use in articular cartilage repair and potentially for growth plate cartilage repair. In addition, MSCs have also been documented as possessing unique immunosuppressive properties which are advantageous during procedures such as transplantation [57, 58]. Furthermore, it has been hypothesized that bone marrow-derived MSCs secrete various factors which are bioactive with the ability to inhibit scar tissue formation, suppress apoptosis, stimulate angiogenesis [59], as well as having immunoregulatory and regenerative properties [60–62] in comparison to MSC derived from other sources.

 Using a growth plate injury model in rabbits, Chen et al. [63] successfully transplanted periosteum-derived MSCs into the growth plate defect and found that the high-proliferation rate of MSCs made them an excellent source for donor cells [63]. Similar to chondrocyte transplantation, two potential methods of delivering MSCs into the desired area of injury is via autologous or allogeneic transplantation. Autologous transplantation of MSCs involves the harvesting of patients' own MSCs and then reimplantation after *ex vitro* expansion. On the other hand, allogeneic transplantation of MSCs involves the use of MSCs taken directly from a cell bank. Planka et al. [64] compared the differences between autologous and allogeneic MSC transplantation and found that there were no major differences in the effect of these implanted MSCs on tibia length and potential angular deformities [64]. Furthermore, the implantation of these cells saw the formation of hyaline chondrocytes within the growth plate injury site [64]. This result was also seen when allogeneic MSCs were transplanted into the site of growth plate injury in a guinea pig model [65].

It is not guaranteed that implanted MSC will change into the desirable chondrocytes. The differentiation of MSCs is highly dependent on cellular environment, hence, is heavily influenced by the presence of certain growth factors [66]. To optimise the expansion and chondrogenesis of MSCs for cartilage repair, certain growth/survival, and chondrogenic factors need to be present in order to stimulate the migration, growth, survival and chondrogenic potentials of MSCs or progenitor cells. There have been many previous studies which have identified some stimuli or signal molecules controlling their migration, proliferation (PDGF-BB, *FGF-2*), and chondrogenic differentiation (TGF-*β*1, IGF-I). PDGF-BB has long been found to be an important growth and survival factor of MSCs [54, 67], and *FGF-2* has been shown to enhance mitotic and chondrogenic potentials of human bone marrow-derived MSCs in culture [68]. TGF-*β*3 has been shown to stimulate chondrogenic differentiation in MSCs and expression of cartilage matrix molecules [69, 70]. In support, Anh et al. [71] found that in young New Zealand White rabbits with growth plate defects, gelfoam (porcine skin gelatin) with MSC, as well as TGF-*β*3 was found to have remarkably reduced the angular deformity following injury repair [71]. On the other hand, one more recent study which used similar methods in an ovine tibial growth plate injury model did not produce successful cartilage regeneration outcome at the injured growth plate (which is in contrast to the their rabbit model) [72]. However, the study found the addition of the MSCs/growth factor/gelfoam complex did not alter the rate of bony repair formation [72]. In addition, IGF-I has been found to be essential for the differentiation and maturation of growth plate chondrocytes, with important anabolic effects on matrix production for maintaining articular cartilage homeostasis. Furthermore, it has been shown that the structural, functional, and molecular properties of engineered cartilage can be modulated by sequential application of growth factors [54]. While TGF-*β* stimulates MSC chondrogenesis and IGF-I can enhance their extracellular matrix synthesis, the combined stimulatory effects of TGF-*β*1 and IGF-I may form a potentially valuable dual stimulatory effect on intrinsic or transplanted MSC function. Such combined stimulatory effects have been demonstrated in the chondrogenesis of periosteum MSCs *in vitro *[69]. Overall from the few studies which focus on combined effects of growth factors and MSC implantation, supplementation with an appropriate growth factor or combination of growth factors is important for a successful outcome for MSC-based growth plate cartilage regeneration. However, further studies are required exploring potential, more potent growth factors, their optimal delivery and formulation for enhancing success for MSC use in cartilage engineering. 

Without the correct support and environment, studies have shown that newly injected MSCs were not able to be viable for a sufficient length of time [73]. Therefore, similar to chondrocyte transplantation, to increase longevity and activity, to encourage chondrogenesis as well as to direct the transplanted MSCs into the desired area, a supporting scaffold made from an appropriate material is needed. A myriad of natural and synthetically produced materials have been studied such as fibrinogen, collagen, collagen derivatives, as well as various man-made polymers and other synthetic biomaterials. Di Martino et al. [74] outlined several important qualities when developing the ideal scaffold including biocompatibility, bioabsorbability/ biodegradability, appropriate pore size, as well as providing a stable foundation for new tissue formation—in particular suitable for MSC growth, proliferation, and chondrogenesis [74]. Currently, many studies which have reported success in MSC transplantation and differentiation use scaffolds of various types made of natural substances. Natural substances are biologically more compatible and biodegradable, and they provide a more natural microenvironment for the embedded MSCs [59]. Some of the commonly used natural materials are both protein and carbohydrate-based, including chitosan, collagens, fibrin gels, hyaluronan, and alginate [47, 75–80].

Some of the more commonly researched natural materials for use in cartilage regeneration in growth plate and articular cartilage studies include chitosan and fibrin gels. Planka et al. [64] embedded MSC into a scaffold of chitosan and collagen and placed the complex into the growth plate injury site of miniature pigs. The gel scaffold was able to be sealed with a bioceramic material to stop cells from deviating from the desirable area which resulted in some success in preventing growth arrest and angulation deformity [65]. Similarly, an earlier study conducted by Li et al. [81] reported their chitosan-MSC construct was able to restore large growth plate defects in immature rabbits [81]. Medrado et al. [82] also reported the benefits of a chitosan-gelatine construct *in vivo*, whereby the addition of MSCs and dexamethasone resulted in an increase of cell adhesivity, proliferation as well as cell viability. Interestingly, the addition of dexamethasone found an increase in the concentration of collagen-2a when combined with the chitosan-gelatine-MSC complex [82]. Apart from chitosan, a few studies have used materials such as agarose—a polysaccharide obtained from agar. Chen et al. [63] did a large growth plate defect study on a 6-week-old NZW rabbits using agarose with embedded MSCs harvested from the periosteum. Chen et al. [63] found that growth arrest and angular deformation and loss of length of tibia induced by the growth plate defect were corrected by the MSC-agarose treatment in comparison to agarose-only controls [63]. In addition to these naturally occurring substances, synthetic materials such as poly (lactic-co-glycolic acid) (PGLA) and poly (lactic acid) (PGA) have also been used for cartilage tissue engineering. Unlike chitosan and other natural substances, these synthetic counterparts allow modifications such as pore size, fibre diameter, and degradation properties to suit their specific use. Previous studies have found some success in using these synthetic materials for articular cartilage regeneration [83–85]. However, some of the limitations associated with their use include relatively poor cell adhesion properties as well as issues concerning their biocompatibility [86]. 

In recent times, the development of injectable hydrogels has become of great interest for cartilage repair and potentially growth plate cartilage regeneration. These are gel-like substances which can have MSC embedded into them [87]. Hydrogels offer the administration of growth factors and/or cells into a cartilage defects more accessible and easier. Cho et al. [88] have developed alginate/polyvinyl alcohol (PVA) hydrogels which is able to gelatinize at a more controllable rate than solely alginate hydrogels. Future studies will reveal potential of MSCs alongside these natural and injectable scaffolds and the appropriate growth factors to regenerate articular and growth plate cartilage.

## 6. Combined MSC and Gene Therapy Approach for Cartilage Repair

Successful chondrogenic regeneration involves two key points—first, to encourage chondrogenesis, and second to form new cartilage. Although current studies have provided a myriad of different bioactive factors that have potential to greatly benefit the repair process, difficulties associated with their administration have slowed down any real progress. This explains why new techniques involving methods such as genetic engineering and gene transfer technology have become of interest. Although a majority of these studies were not done specifically for repairing growth plate cartilage, many of the techniques could potentially be applied for this use. Successful gene transfer can be achieved through a few different approaches: the direct vector administration to cells or surrounding cells within the injury site or alternatively and the transplantation of genetically modified chondrogenic cells into the affected area [89]. 

Direct modification of *ex vivo* chondrocytes has been well studied. Cultured chondrocytes were able to maintain the expression of certain transgene products after genetic modification with recombinant adenoviral of TGF-*β* [90, 91], BMP-7 [92], and IGF-I [91, 93]. Nixon et al. [93] found that *in vitro* experiments involving the adenoviral over expression of IGF-I in chondrocytes resulted in the stimulated expression of proteoglycans as well as collagen type 2 [93]. Proteoglycan and collagen type 2 synthesis were also stimulated when TGF-*β*1 was transduced adenovirally on a monolayer of chondrocytes [90, 91]. In more recent times, interest has been shown for the gene transfer of transcriptional factors such as Sox-9. Sox-9 is a known master regulator of chondrogenesis, hence when Sox-9 was retrovirally overexpressed, it resulted in increased collagen type 2 expression in a pellet culture [94]. 

Since treatment with growth factors is often not successful due to the short half-life of many growth factors, and since gene delivery is a better alternative to deliver growth factors because it is more stable and flexible than the protein itself [95], much interest has been drawn towards genetic modification of MSCs with growth factor genes for enhancing cartilage repair. This technique requires the *ex vivo* genetic modification of MSCs followed by the transplantation of the altered cells back into the affected area [59]. Since modification by any means is an alteration of the original, Hu et al. [96] questioned whether gene-altered MSCs were still capable of possessing their characteristic of multipotency [96]. They found that after retroviral transfection with human IGF-I, rat MSCs showed a greater ability to express IGF-I as well as an increased ability to proliferate and reduce apoptosis, and that modifications of MSCs could potentially affect the types of tissues they differentiate into [96]. In order to lengthen the time and versatility of MSCs, Song et al. [97] utilised gene therapy to transfect bone marrow-derived MSCs with the *FGF-2* gene, which showed an improvement in survival of MSC against hypoxic conditions *in vitro* [97]. In addition, another study also modified MSCs with angiogenin adenoviral vector which resulted in the enhancement of implanted cells against hypoxic injury [98]. 

Although there are many studies which have successfully transduced MSCs with variety of chondrogenic growth factors, an interesting study discussed some of the limitations of this MSC + gene therapy approach for cartilage repair. A study by Palmer et al. [99] showed that only a certain amount of gene expression was needed to induce chondrogenic differentiation of bone marrow-derived cells, and that overexpression by gene-induced transduction may have negative, opposing effect on chondrogenic differentiation [99]. In addition, a few other disadvantages of the *ex vivo* approach for gene therapy include high cost and being fairly laborious and time consuming. However, *ex vivo* gene therapy allows the safety testing and control of the cells before the reimplantation and hence minimising any risk of disease transmission [100]. Overall, although the potential of combining gene therapy techniques with MSCs has been more recently explored, not many have applied it to the regeneration of growth plate cartilage. Hence, further studies are needed to investigate whether this type of cartilage engineering is useful in growth plate cartilage regeneration.

## 7. Endogenous Stem Cell Possibility

Although a number of studies with rabbit growth plate injury repair models have shown that MSCs may have some potential in regenerating injured growth plate and prevent bone growth defects [63–65, 71]. However, recent work with a large animal model has questioned value of this *ex vivo* MSC approach [72]. While ovine bone marrow MSCs are multipotential and can form cartilage-like tissue *in vivo* [101], however, in a growth plate injury model in lambs, autologous bone marrow-derived *ex vivo*-expanded MSCs failed to promote growth plate regeneration [72]. In addition, currently, MSC-mediated cell therapies are limited by various difficulties and issues such as morbidity associated with cell harvest, difficulty in stem cell isolation, genetic and phenotypic instability associated with *ex vivo* expansion, difficult up-scaling, high costs, variability, and risks of disease transmission particularly with allogeneic MSC transplantation [54]. While transplantation of both allogeneic and autogenous MSC as well as modified MSCs offer many advantages in cartilage repair, a major problem associated with their use is the need for fetal calf serum during *ex vitro* expansion. 

The existence of functional stem cells within the local environment and their migratory capacity represent an opportunity to circumvent limitations of *ex vivo*-based MSC therapy and to achieve *in situ* cartilage regeneration by enhancing local reparative mechanisms and mobilising endogenous MSCs [52, 102]. MSCs express adhesion molecules [103] and can migrate to sites of injury healing [51, 97]. Indeed, synovial mesenchymal cells migrate to cartilage defects and may serve as a cell source for repair under specific growth factor stimulation [104], and marrow MSC migrate and contribute to cartilaginous formation during bone healing and contribute to articular repair [105]. 

Although present in small quantities during the fibrogenic infiltrate with the growth plate injury site, endogenous multipotent mesenchymal stromal cells were observed during growth plate injury repair [106]. These cells demonstrated their multipotency—differentiating into bone and cartilage tissues within the injury site [67, 106–110]. With infiltration of progenitor cells into growth plate injury site, it will be of particular interest to investigate whether these endogenous progenitor cells can be mobilised to enhance growth plate regeneration. 

However, the main problem with accessing the endogenous cells for example from the bone marrow is that they may not be present in a density large enough to support adequate cartilage regeneration. Hence, to overcome this problem, a recent study has suggested that selected growth factors are needed to stimulate and enhance MSC migration and accumulation into the cartilage injury site [111]. Dar et al. [112] found the chemokine/receptor pair SDF-1/CXCR4 is present and functional in MSC population [112]. Kitaori et al. [113] found that inhibition or absence of this signaling resulted in absence of MSC in a bone fracture model [113]. In addition to the SDF-1/CXCR4 signalling, Schenk et al. [114] reported monocyte chemotactic protein (MCP-3) as another homing chemotactic signaling pathway for MSC migration. Schenk et al. [114] found that in myocardial infarction, the systemic infusion of this protein resulted in a MSC migration response. Furthermore, Ode et al. [115] studied roles and influences of different extracellular matrix components on MSC migration and behavior and found that the collagen family excluding collagen-V as well as adhesion proteins such as fibronection and vitronectin all influenced and encouraged the migratory and proliferatory behavior of MSCs [115]. More studies are required to study how endogenous MSCs can be regulated to enhance migration into and expansion within the injury site for cartilage regeneration.

## 8. Conclusion

Growth plate injuries are common in children and their “faulty” bony repair impairs bone growth and cause life-long orthopaedic problems. Current treatments (surgical correction) for these problems are highly invasive and often requiring repeated surgeries, and thus there is a strong need for a biological treatment that can promote growth plate cartilage regeneration. Although the approach of using *ex vivo* expanded MSC has shown some promise in promoting growth plate repair in rabbit models, the efficacy of this approach has been questioned in a recent “translational study” using a large animal model. Further studies are required to define more potent chondrogenic growth factor(s) or matrix scaffold that will enhance growth plate regeneration using *ex vivo*-expanded MSC, and more studies are needed to investigate the therapeutic potential of MSCs for growth plate regeneration in large animal models. In addition, due to the time (around 3 weeks) required for MSC isolation and expansion, this *ex vivo* approach with autologous MSC may not be practical to treat a growth plate shortly after the fracture; further studies are required to investigate whether endogenous MSCs or progenitor cells within the local environment or bone marrow can be mobilised and local regenerative mechanisms be optimised to achieve *in situ* growth plate regeneration after a growth plate fracture so to circumvent limitations of *ex vivo*-based MSC therapy.

##  Conflict of Interests

All authors declare no conflict of interests.
